# SARS-CoV-2 Virus and Human Leukocyte Antigen (HLA) Class II: Investigation in silico of Binding Affinities for COVID-19 Protection and Vaccine Development

**DOI:** 10.29245/2578-3009/2020/4.1198

**Published:** 2020-11-16

**Authors:** Spyros S. Charonis, Effie-Photini Tsilibary, Apostolos P. Georgopoulos

**Affiliations:** 1 Brain Sciences Center, Department of Veterans Affairs Health Care System, Minneapolis, MN 55417, USA; 2 Department of Neuroscience, University of Minnesota Medical School, Minneapolis, MN 55455, USA

**Keywords:** SARS-CoV-2, SRAS-Cov-2 spike protein 2, Human Leukocyte Antigen (HLA), Vaccines

## Abstract

SARS-CoV-2 causes COVID-19, urgently requiring the development of effective vaccine(s). Much of current efforts focus on the SARS-CoV-2 spike-glycoprotein by identifying highly antigenic epitopes as good vaccine candidates. However, high antigenicity is not sufficient, since the activation of relevant T cells depends on the presence of the complex of the antigen with a suitably matching Human Leukocyte Antigen (HLA) Class II molecule, not the antigen alone: in the absence of such a match, even a highly antigenic epitope in vitro will not elicit antibody formation in vivo. Here we assessed systematically in silico the binding affinity of epitopes of the spike-glycoprotein to 66 common HLA-Class-II alleles (frequency ≥ 0.01). We used a sliding epitope window of 22-amino-acid-width to scan the entire protein and determined the binding affinity of each subsequence to each HLA allele. DPB1 had highest binding affinities, followed by DRB1 and DQB1. Higher binding affinities were concentrated in the initial part of the glycoprotein (S1-S460), with a peak at S223-S238. This region would be well suited for effective vaccine development by ensuring high probability for successful matching of the vaccine antigen from that region to a HLA Class II molecule for CD4^+^ T cell activation by the antigen-HLA molecule complex.

## Introduction

SARS-CoV-2 causes COVID-19, a disease that has now become a global pandemic^1,2^. Hence, there is an urgent need to develop an effective vaccine against the virus. Many efforts in that direction focus on the SARS-CoV-2 spike-glycoprotein, seeking to identify highly antigenic epitopes as good vaccine candidates^3^. However, high antigenicity is not sufficient, since the activation of relevant T cells depends on the presence of the complex of the antigen with a suitably matching Human Leukocyte Antigen (HLA) Class II molecule, not the antigen alone: in the absence of such a match, even a highly antigenic epitope in vitro will not elicit antibody formation in vivo. Therefore, there are two key factors for a vaccine to be effective: first, there should be a good match with a HLA Class II molecule, and second, the antigen should be adequately immunogenic, assuming that the individual is immunocompetent for antibody production. In the case of an actual viral infection, the mounting of antibodies against the virus protein is called “specific (or adaptive) immunity”. The key agents for specific immunity and antibody production are the HLA Class II molecules. These bind to ~18–22-mer AA peptides resulting from the cleavage of the viral proteins by proteases in antigen presenting cells (APC), which include macrophages^4^. When a match occurs, the HLA Class II-peptide complex moves to the cell surface, attracts CD4^+^ T cells, and a complex process of antibody production is initiated^5^. Thus, the most crucial first important step is the successful match between the virus protein peptide and a Class II molecule. An individual carries 6 classical Class II alleles (2 from each DRB1, DQB1 and DPB1 genes)^4^. If these provide a good match to the peptide, the antibody production process starts immediately and, assuming that the person is immunocompetent, antibodies will be produced in time to eliminate the virus and protect from future exposures to that virus. If, on the other hand, there is no match, antibodies cannot be produced and there will not be specific immunity for that virus. In fact, complete lack of Class II molecules, as it occurs in a rare genetic disorder (primary immunodeficiency disorder^6^) is a devastating disease characterized by uncontrolled viral infections and poor prognosis. Finally, the possibility and quality of the match critically depend on the affinity of the virus peptides and the HLA Class II molecule. Indeed, in this study, we examined systematically and exhaustively the SARS-CoV-2 virus spike glycoprotein to determine the binding affinities of epitopes to 66 common HLA Class II alleles and then identify regions in the protein with high such affinities as good candidates for vaccine development. The structure of the spike protein (S) of SARS-CoV-2 has been recently solved using cryo-electron microscopy^7^ and its sequence is known^8^. Specific attention has been directed towards the spike glycoprotein of the SARS-CoV-2 since it represents a major binding site for cells via their ACE2 receptors in a number of recent studies that even revealed its crystal structure^2,9,10^.

## Materials and Methods

The main objective of this study was to exhaustively assess the binding affinities of HLA Class II molecules to the SARS-CoV-2 spike glycoprotein. For that purpose, we assessed the binding affinities of 66 Class II alleles, as follows.

### HLA alleles

For this study, we selected the more frequent alleles of classical HLA Class II genes (DPB1, DQB1, DRB1), namely all alleles with global frequencies ≥ 0.01, an arbitrary but reasonable threshold. For that purpose, we obtained an Estimation of Global Allele Frequencies by querying the website http://www.allelefrequencies.net/ ^11^. The alleles with frequencies ≥ 0.01 that we used are given in Table 1. They comprised 21, 15 and 30 alleles of DPB1, DQB1 and DRB1 genes, respectively.

#### SARS-CoV-2 spike glycoprotein

The amino acid (AA) sequence of the SARS-CoV-2 spike glycoprotein (“glycoprotein”) was retrieved from the UniprotKB database^12^. It consists of 1273 AAs.

#### Partitioning the SARS-CoV-2 spike glycoprotein

**Figure. 1.**
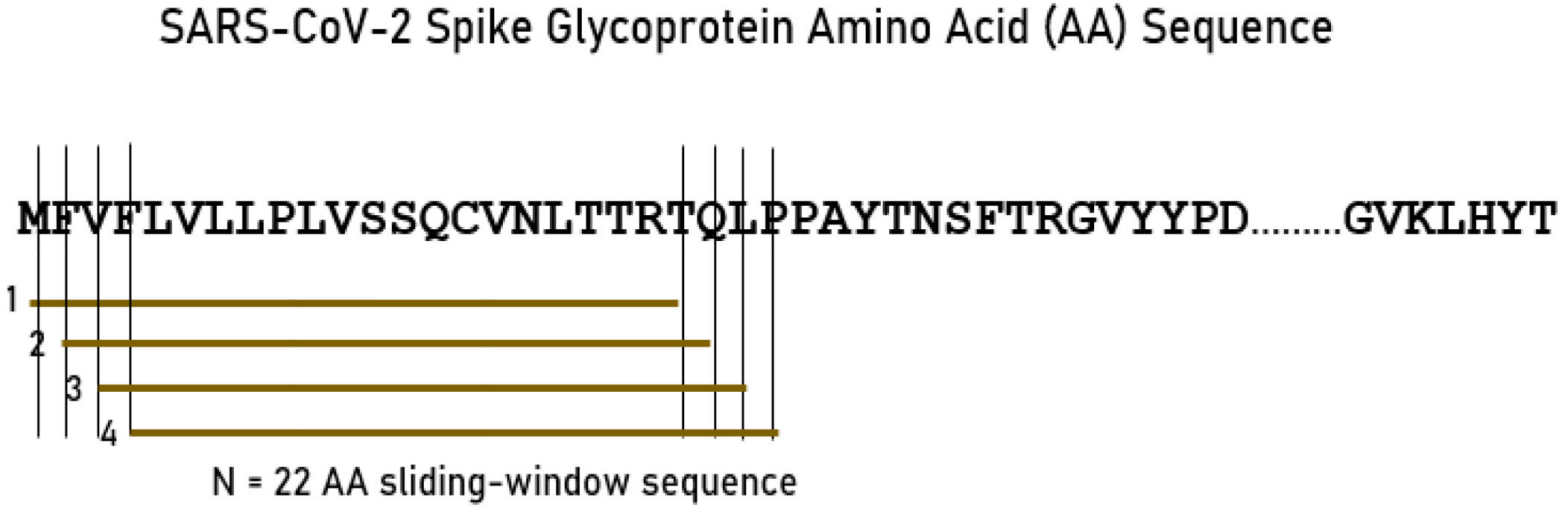
A sample of the sliding window approach for the SARS-CoV-2 spike glycoprotein.

As mentioned above, the main objective of this study was to assess exhaustively the binding affinities of HLA Class I and II molecules to the SARS-CoV-2 spike glycoprotein. For that purpose, we used a sliding epitope window approach^13^ to partition the sequence of the spike glycoprotein into subsequences of all possible consecutive 22-mers for (e.g. residues S1-S22, S2-S23, …, S1252-S1273) that covered the entire sequence length (1273 AA). The method is illustrated in Figure 1. More specifically, a set of 22-AA-length subsequences was generated (number of subsequences = length of spike glycoprotein – 22); thus the number of subsequences was 1273–22 = 1251. Subsequences were collected and queried in the IEDB database (www.iedb.org)^14^ in order to determine their binding affinity to a specific HLA Class II molecule. Binding affinity predictions were obtained using the NetMHCIIpan method^14^. For each *22*-mer, a binding affinity score was predicted and reported as a percentile rank by comparing the peptide’s score against the scores of five million random *n*-mers selected from the SwissProt database^11^. Smaller percentile ranks indicate higher binding affinity. Therefore, the minimum percentile rank (i.e. highest affinity) for each allele and *22*-mer of the spike glycoprotein was found and kept. This yielded 1251 values for each allele.

### Data analysis

We used two measures to quantify the binding affinity of an allele to the glycoprotein: (a) the value with the lowest *22*-mer minimum rank (of the total 1251 values), called Lowest Percentile Rank (LPR), and (b) the percent of percentile rank values less or equal 5% (PR5). LPR is a unique value, and, therefore, prone to chance fluctuations, whereas PR5 is a more stable measure with the reasonable interpretation as the probability of high binding affinity of the allele at the 5% threshold. (Exploratory analyses using a 10% threshold yielded very similar results.) The data were then analyzed to assess (a) the effect of Gene on LPR and PR5, and (b) the differential variation of Gene-related effect on LPR and PR5 along the AA sequence of the glycoprotein to determine epitopes of high binding affinity to specific alleles.

#### Analyses using bootstrap

Since each individual carries 6 HLA Class II alleles of the classical genes (two of each DPB1, DQB1, DRB1), we simulated by bootstrap sets of individual cases by combining randomly six alleles (two from each of the three genes, DPB1, DQB1, and DRB1) using ad hoc computer programs in Microsoft Visual Intel FORTRAN (version 2019).

##### Application to single individuals.

As mentioned above, every individual carries 6 classical HLA Class II alleles, 2 from each gene (DPB1, DQB1, DRB1). For a specific individual k, a quantitative estimate ${\text{Z}}_{k}$ of their ability to make antibodies against SARS-CoV-2 would be the average PR5 of the 6 alleles carried by that individual. Let $\zeta_{i}$ be the PR5 for allele i. Then

(1) $${\text{Φ}}_{k}=\sum \zeta_{1}^{DPB1}+\zeta_{2}^{DPB1}+\zeta_{1}^{DQB1}+\zeta_{2}^{DQB1}+\zeta_{1}^{DRB1}+\zeta_{2}^{DRB1}$$

and

(2) $$\bar{\zeta_{k}}=\frac{{\text{Φ}}_{k}}{6}$$

We estimated basic statistics for $\bar{\zeta_{k}}$ using a bootstrap procedure, as follows. Let $M_{\text{DPB1}}$, $M_{\text{DQB1}}$, $M_{\text{DRB1}}$ be the number of available alleles in the DPB1, DQB1 and DRB1 genes, respectively, in the population; in this study, for the most common (f≥0.01) alleles globally, we have: $M_{\text{DPB1}}=21$, $M_{\text{DQB1}}=15$, and $M_{\text{DRB1}}=30$. We generated 1000 bootstrap $\bar{\zeta_{k}^{*}}$ values by selecting randomly (with replacement, allowing for homozygotes) two alleles from each set of the three genes

(3) $${\text{Φ}}_{k}^{*}=\sum \zeta_{1*}^{DPB1}+\zeta_{2*}^{DPB1}+\zeta_{1*}^{DQB1}+\zeta_{2*}^{DQB1}+\zeta_{1*}^{DRB1}+\zeta_{2*}^{DRB1}$$

and

(4) $$\bar{\zeta_{k}^{*}}=\frac{{\text{Φ}}_{k}^{*}}{6}$$

where the asterisk (∗) denotes a bootstrap sample.

##### Application to populations.

Here we extended the analysis above on single individuals to populations, by taking into account the frequency $f_{i}$ of allele i in the population. For that purpose, we computed an estimate ${\text{Z}}_{k}^{*}$ of the weighted (by $f_{i}$) binding affinity $\zeta_{i}$ for an individual k in the population using the bootstrapping procedure above:

(5) $${\text{Z}}_{k}^{*}=\sum f_{1k^{*}}^{DPB1}\zeta_{1k*}^{DPB1}+f_{2k*}^{DPB1}\zeta_{2k*}^{DPB1}+f_{1k*}^{DQB1}\zeta_{1k*}^{DQB1}+f_{2k*}^{DQB1}\zeta_{2k*}^{DQB1}+f_{1k*}^{DRB1}\zeta_{1k*}^{DRB1}+f_{2k*}^{DRB1}\zeta_{2k*}^{DRB1}$$

where the asterisk (∗) denotes a bootstrap sample. We then computed the sum of the corresponding frequency weights:

(6) $$F_{k}^{*}=\sum f_{1*}^{DPB1}+f_{2*}^{DPB1}+f_{1*}^{DQB1}+f_{2*}^{DQB1}+f_{1*}^{DRB1}+f_{2*}^{DRB1}$$

and

(7) $$\bar{f_{k}^{*}}=\frac{F_{k}^{*}}{6}$$

We then computed the mean $\bar{\text{Z}}$ of weighted ζ as follows (N=1000 bootstrap samples).


(8) $${\text{Z}}^{\prime }=\sum_{k}^{k=1,N}Z_{k}^{*}$$

(9) $${\text{F}}^{\prime }=\sum_{k}^{k=1,N}{\text{F}}_{k}^{*}$$

(10) $$\bar{\text{Z}}=\frac{{\text{Z}}^{\prime }}{{\text{F}}^{\prime }}$$

##### Association between binding affinity and allele frequency in populations.

Here we investigated the possible association between the average binding affinity $\bar{\zeta_{k}^{*}}$ (equation 2) and the corresponding average allele frequency $\bar{f_{k}^{*}}$ (equation 7) using a linear regression analysis.

As a control, we paired the observed $\bar{\zeta_{k}^{*}}$ to randomly chosen $\bar{f_{k}^{*}}$ (from the same gene set) and performed the same analysis.

##### Association between binding affinity and allele frequency in single genes.

Here we investigated the possible association between allele frequency and binding affinity (PR5) for each one of the 3 genes above using a linear regression analysis.

#### General statistical methods

Standard statistical methods were employed in these analyses, including analysis of variance (ANOVA) and linear regression, using the IBM-SPSS statistical package (version 26).

## Results

### Overall relations of individual HLA Class II alleles to SARS-CoV-2 spike glycoprotein

**Figure. 2.**
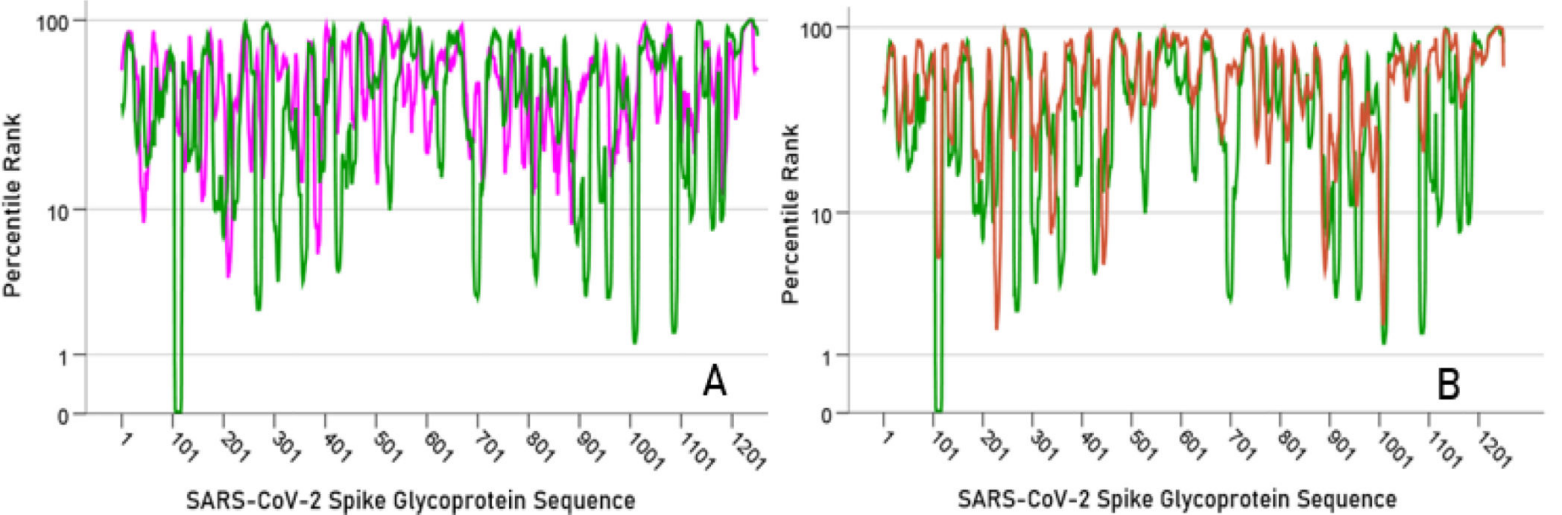
**A)** percentile rank values of all 22-mer sliding epitopes (N = 1251) are plotted along the SARS-CoV-2 glycoprotein sequence for DRB1:13:02 (green; low rank values = high binding affinities) and DQB1*02:02 (magenta; high rank values = low binding affinities). **B)** all percentile rank values are plotted for 2 alleles that differ by only one amino acid and yet possess very different binding affinities (DRB1*1301, red; DRB1*13:02, green)

**Figure. 3.**
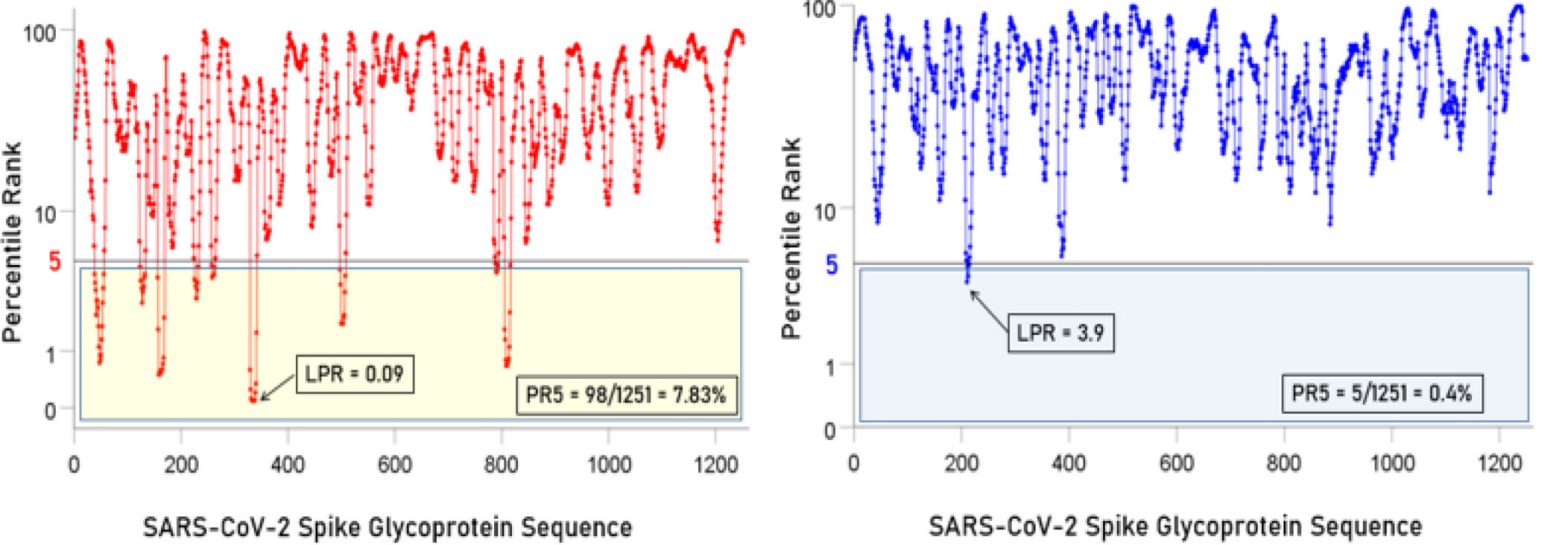
Left panel, percentile rank values and associated LPR and PR5 values for an allele (DPB*04:01) with high binding affinities. Right panel, percentile rank values and associated LPR and PR5 values for an allele (DQB1*02:02) with low binding affinities.

**Figure. 4.**
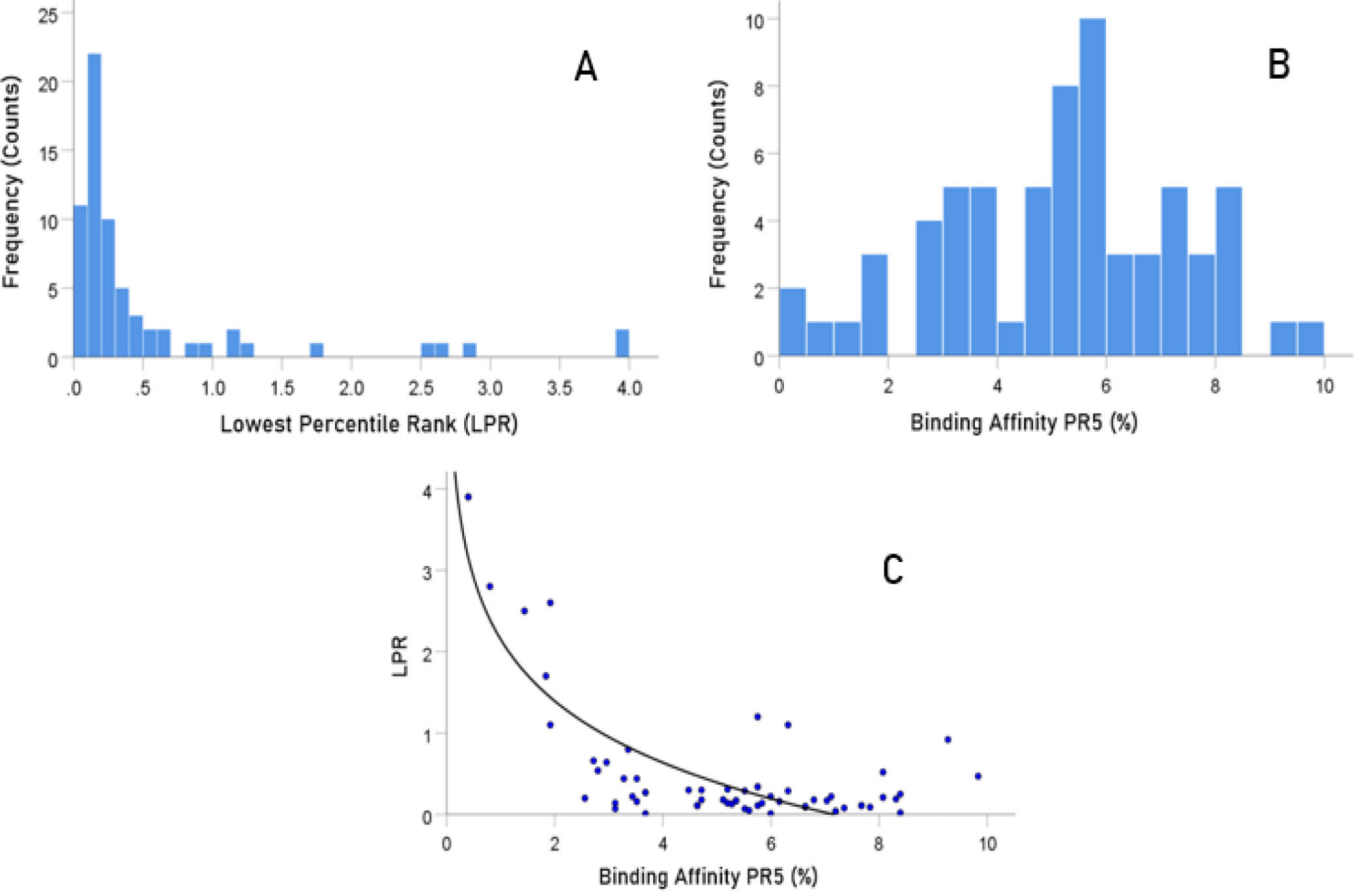
A) frequency distribution of LPR. B) frequency distribution of PR5. C) LPR is plotted against LE5. See text for details.

Figure 2 illustrates the results of the sliding epitope window analysis for 3 HLA Class II alleles. Fig. 2A shows the percentile rank values for all 1251 epitopes for an allele with high binding affinities (DRB1*13:02) and another with low binding activities (DQB1*02:02). Fig. 2B plots the percentile rank values for 2 HLA Class II alleles that differ only by one amino acid (DRB1*13:01, DRB1*13:02) and attests to the importance of HLA Class II molecule structure on binding affinity to SARS-CoV-2: DRB1*13:02 has much higher binding activities than DRB1*13:01. Figure 3 illustrates results on LPR and PR5 for two alleles, one with high (left panel) and another with low (right panel) binding affinities. Figure 4 shows the frequency distributions of LPR (Fig. 4A) and PR5 (Fig. 4B), and their joint distribution (Fig. 4C). LPR and PR5 were significantly associated; more specifically, PR decreased with the logarithm of PR5 (linear regression of LPR against ln(PR5), F_[1,64]_ = 145.2, P = 4.1×10^−18^). Tables 2 and 3 give the LPR and PR5 values of the 66 alleles studied.

### Overall relations of individual HLA Class II genes to SARS-CoV-2 spike glycoprotein

**Figure. 5.**
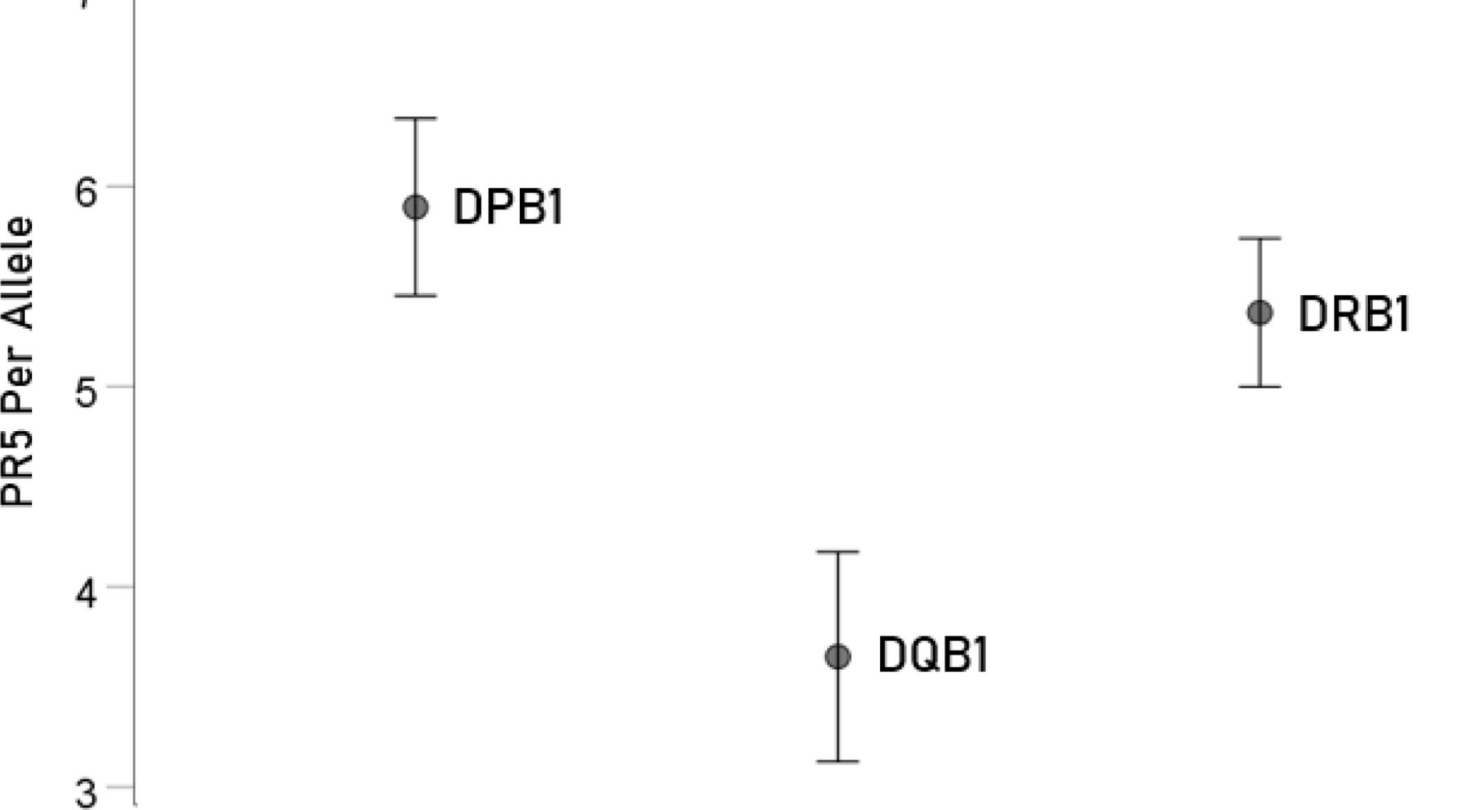
Mean ± SEM of percent of PR5 for the 66 alleles used (N = 21, 15, 30 for genes DPB1, DQB1 and DRB1, respectively). See text for details.

The PR5 of the three genes (DPB1, DQB1, DRB1) differed significantly (Figure 5; F_[2,63]_ = 5.69, P = 0.005, ANOVA). DPB1 had significantly higher PR5 than DQB1 (P = 0.002, ANOVA); the PR5 did not differ significantly between DPB1 and DRB1 (P = 0.365, ANOVA). Finally, DRB1 had significantly higher PR5 than DQB1 (P = 0.009, ANOVA).

### Localization of allele binding affinities along the SARS-CoV-2 spike glycoprotein sequence

**Figure. 6.**
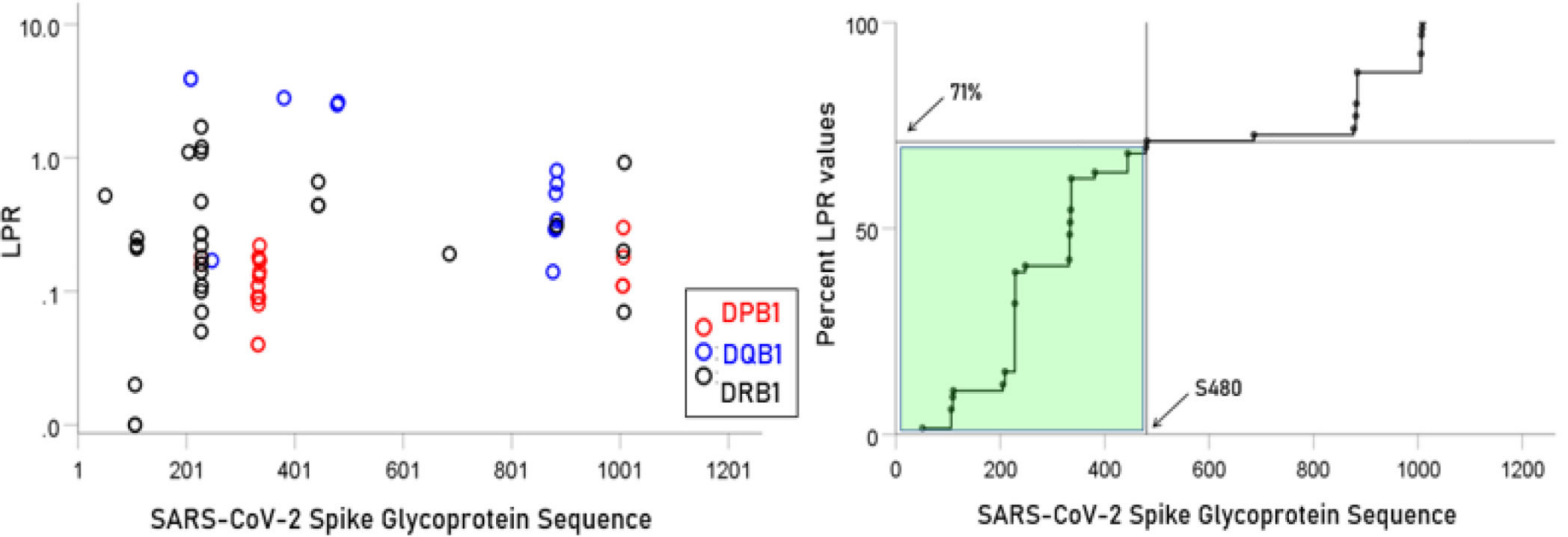
Left panel: LPR for the 66 alleles. Right panel, cumulative plot of the data in the left panel.

**Figure. 7.**
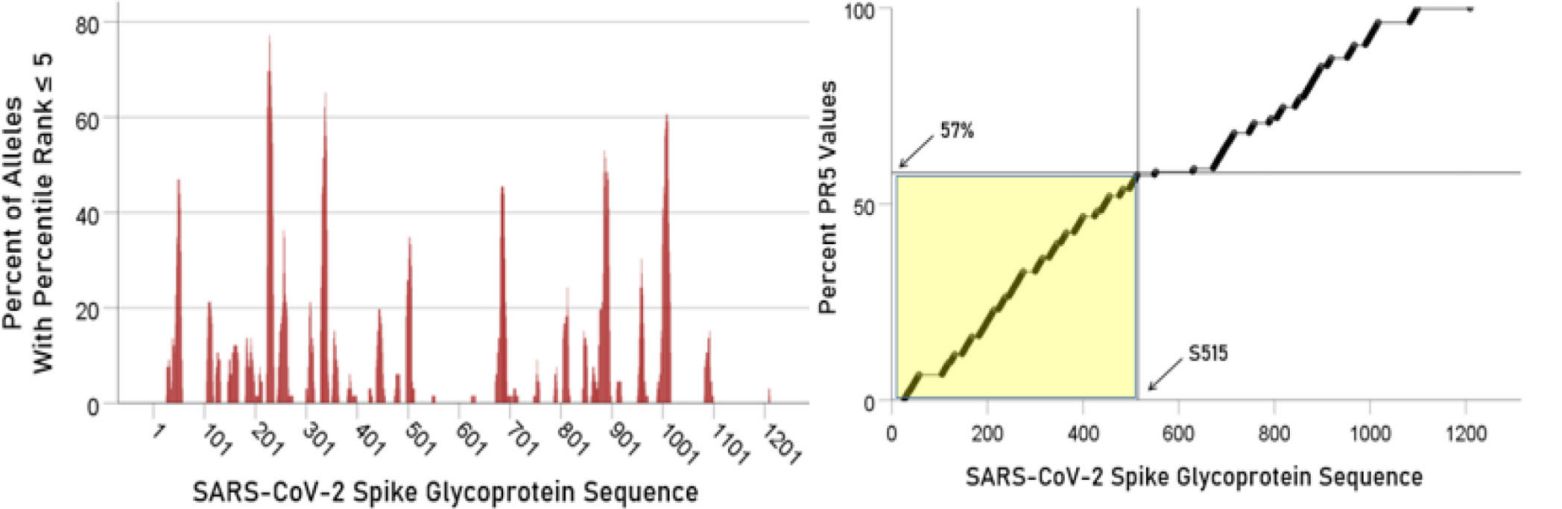
Left panel: Total counts of PR5 values for all 66 alleles used are plotted against the glycoprotein sequence. Right panel, cumulative plot of the data in the left panel.

Lower LPR values (i.e. higher binding affinities) were concentrated in the initial part of the glycoprotein (approximately between S1-S480 AA residues) (Figure 6, left panel); a cumulative plot is shown in Fig. 6, right panel. Similarly, high PR5 values were more frequent in the initial part of the glycoprotein (approximately S1-S501; Figure 7).

### Relevance of allele-SARS-CoV-2 binding affinities to single individuals

**Figure. 8.**
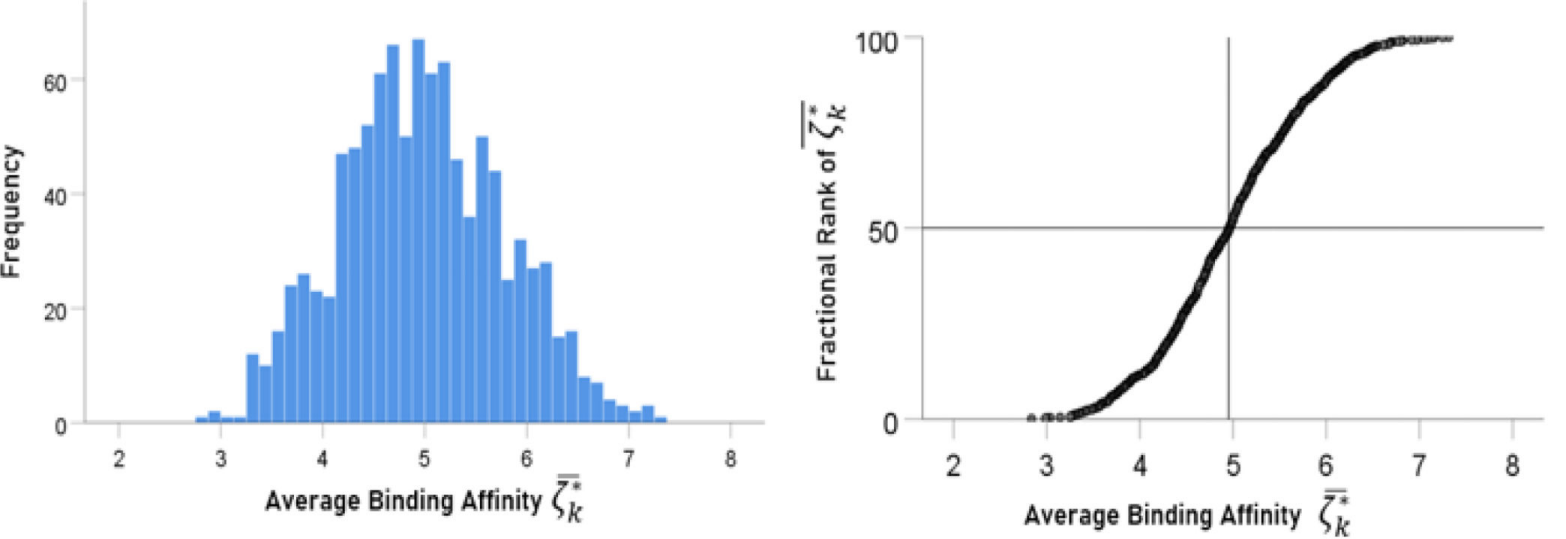
Frequency distribution of 1000 $\bar{\zeta_{k}^{*}}$. See text for details.

The frequency distribution of 1000 average binding affinities $\bar{\zeta_{k}^{*}}$ (see Methods) is shown in Figure 8 (left panel) and their rank-ordered values in Fig. 8 (right panel). The mean and median of $\bar{\zeta_{k}^{*}}$ were 4.97 and 4.96, respectively. These results indicate that ~50% of the individuals in the simulated population possess binding affinities to SARS-CoV-2 spike glycoprotein greater than the average binding affinity of the whole sample.

### Relevance of allele-SARS-CoV-2 binding affinities to whole populations

In this analysis, we extended the findings above on single individuals to populations, by taking into account the frequency $f_{i}$ of allele i in the population by computing an estimate ${\text{Z}}_{k}^{*}$ of the weighted (by $f_{i}$) binding affinity $\zeta_{i}$ for an individual k in the population using the bootstrapping procedure described in the Methods section. We found that $\bar{\text{Z}}=5.01$, a value very close to the mean (= 4.97) and median (= 4.96) of the distribution of $\bar{\zeta_{k}^{*}}$.

In the analyses above, we used the global frequencies of the 66 alleles. However, those frequencies differ from population to population (country, ethnicity, etc.). In general, the quantitative ability to mount antibodies in a population depends (a) on the frequency of those alleles in the population, and (b) on the binding affinity of alleles in the population. Therefore, for accurate estimates of $\bar{\text{Z}}$ in various populations, the frequencies of all HLA Class II alleles in the population should be known.

### Association between binding affinity and allele frequency in populations

**Figure. 9.**
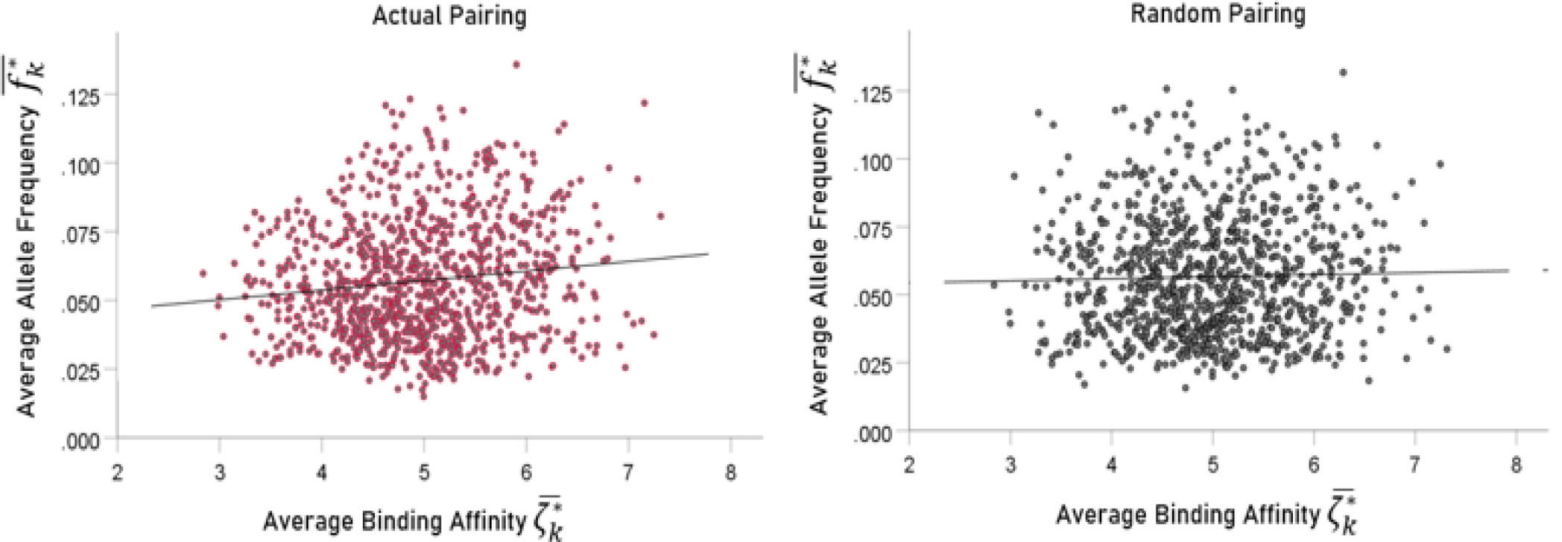
Average six-allele frequency $\bar{f_{k}^{*}}$ is plotted against randomly assigned average binding affinity $\bar{\zeta_{k}^{*}}$ for actual pairing (left panel) and random pairing (right panel). See text for details.

Here we investigated the possible association between the average binding affinity $\bar{\zeta_{k}^{*}}$ (equation 2) and the corresponding average allele frequency $\bar{f_{k}^{*}}$ (equation 7). We found that the average binding affinity $\bar{\zeta_{k}^{*}}$ was positively associated with average allele frequency $\bar{f_{k}^{*}}$: the higher the average binding affinity of the 6-allele set, the higher the average frequency of the alleles in the set (Figure 9, left panel; r = 0.129; slope (beta) ± SE = 0.0348 ± 0.000846 ${\text{t}}_{\left[998\right]}=4.12$, P=0.000042). This means that a 1% increase in $\bar{\zeta_{k}^{*}}$ is associated with an increase of 0.0348 in $\bar{f_{k}^{*}}$. This can be interpreted as reflecting an evolutionary pressure for increasing allele frequency with overall higher affinities to SARS-CoV-2, with a presumed survival advantage. As a control, we paired the observed $\bar{\zeta_{k}^{*}}$ to randomly chosen $\bar{f_{k}^{*}}$ (from the same gene set); no significant association was found (Fig. 9, right panel; r = 0.027, P = 0.389). The results above came from a single bootstrap. We carried out 1000 such bootstraps to obtain a long-term expected estimate of the slope of the dependence of $\bar{f_{k}^{*}}$ on $\bar{\zeta_{k}^{*}}$. The mean slope (± SD) was 0.002815 ± 0.00087 $\bar{f_{k}^{*}}$ per $\bar{\zeta_{k}^{*}}$.

### Association between binding affinity and allele frequency in single genes

**Figure 10.**
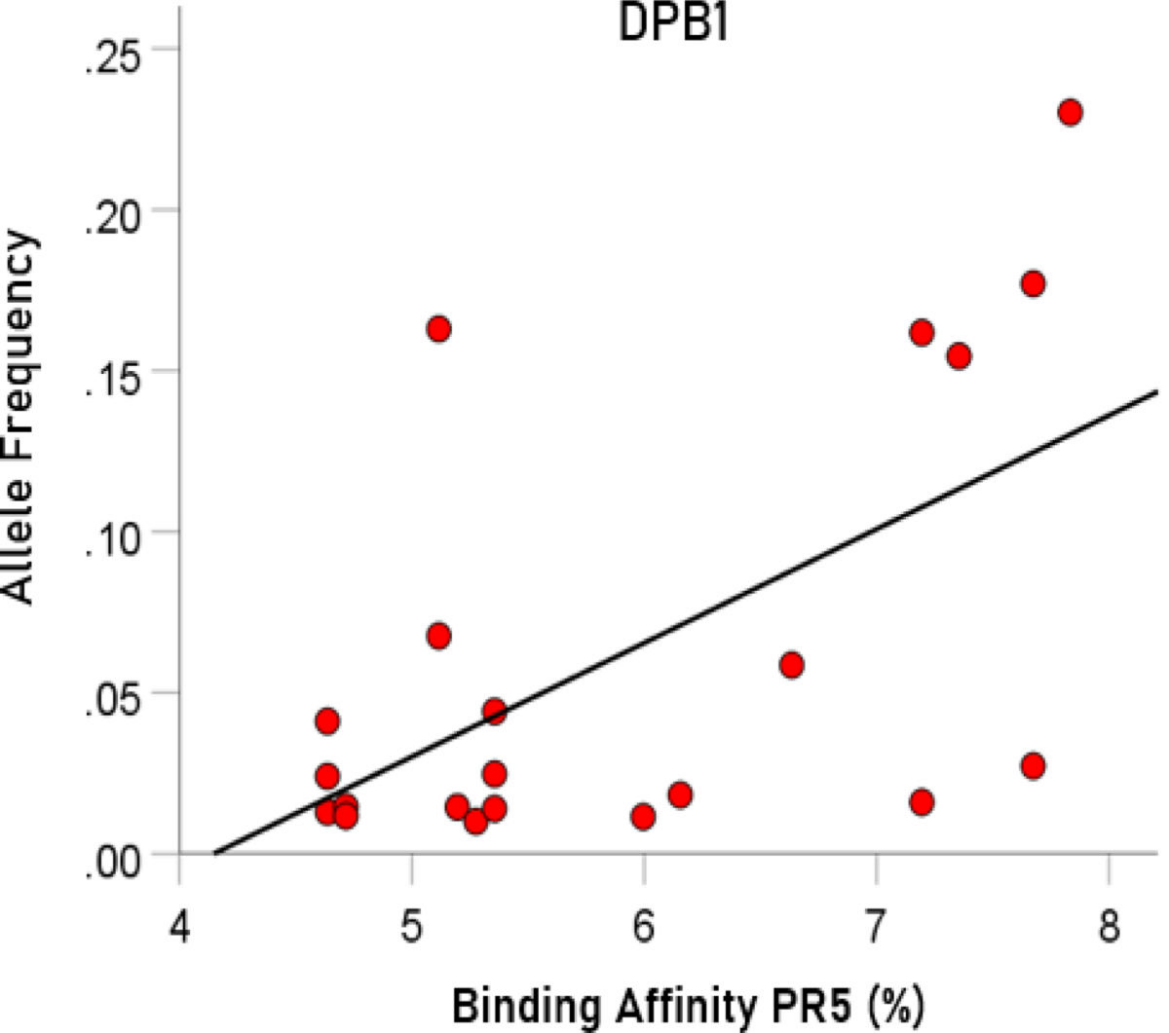
Allele frequency is plotted against binding affinity of corresponding alleles. See text for details.

The results above come from simulated HLA makeup of individuals, consisting of 6 alleles (2 from each gene DPB1, DQB1, DRB1). The significant dependence of average allele frequency on the average binding affinity (Figure 9, left panel) suggests that specific genes might be driving this dependence. We tested this hypothesis by performing a linear regression analysis between allele frequency and binding affinity for each one of the 3 genes above. We found a highly significant correlation only for the DPB1 gene (Figure 10; r = 0.589, P = 0.005, N = 21 alleles). In contrast no significant association was found for the DQB1 gene (r = 0.022, P = 0.938, N = 15) or the DRB1 gene (r = 0.153, P = 0.421, N = 30). These findings indicate that the hypothesized evolutionary pressure has been exerted through the DPB1 gene.

## Discussion

Here we investigated the relations between SARS-CoV-2 spike glycoprotein and 66 common HLA Class II alleles. Our study was inspired by the known dependence of antibody production against a foreign antigen on a good match between the antigen and HLA Class II molecules. There were four major aims of this study, as follows. First, to investigate exhaustively the binding affinity of suitable fragments (epitopes) of the spike glycoprotein to the different 66 HLA Class II alleles; second, to identify portions of the spike glycoprotein with high binding affinity to HLA Class II molecules, as good candidates for vaccine development; third, to derive a quantitative estimate of the ability of an individual to mount antibodies against that protein, as happens during an epidemic or following vaccination; and fourth, to gain an insight into a possible evolutionary pressure by alleles with high binding affinity (and, therefore, the successful production of antibodies and protection from COVID-19) to occur at higher frequencies, i.e. a positive association between binding affinity and allele frequency. We discuss the findings on these objectives below.

### Methodological considerations

The predicted binding affinities were based on an algorithm that considers existing experimental data^14^. Moreover, synthetic peptides which contain amino acid sequences of proteins without post-translational modifications such as glycosylation have been used extensively and have proven an excellent type of molecule for the mimicry of protein sites: such peptides can be generated as exact copies of protein fragments, as well as in diverse chemical modifications^16^. An example of the usefulness of this approach^17^ was the identification of peptides mimicking antibody binding to the Epidermal Growth Factor Receptor.

Studies of binding affinities of HLA Class II molecules to a protein typically focus on specific fragments (epitopes) of the protein, based on a priori considerations of interest to the particular fragment. In contrast, here we sought to assess systematically the binding affinities of all continuous epitopes of the SARS-CoV-2 spike glycoprotein with each of 66 common (frequency ≥ 0.01) HLA Class II alleles. For that purpose, we used a sliding epitope window approach^13^ in which a sliding epitope of 22 AA sequence length (suitable for HLA Class II molecule binding) scanned the whole glycoprotein sequence of 1273 AA, for a total of 1251 epitopes. For each epitope, we determined the minimum rank of its binding to each of the 66 alleles used in this study, as compared to 5 million peptides of the same length; the smaller the minimum rank, the better the binding affinity. This procedure enabled us to (a) evaluate binding affinities of individual alleles, (b) compare binding affinities among the 3 classical HLA Class II genes (DPB1, DQB1, DRB1), (c) identify epitopes with high binding affinity along the glycoprotein sequence, and thus (d) evaluate the ability of individuals and populations carrying these alleles to mount antibodies against the spike glycoprotein, assuming that those epitopes are of adequate antigenicity and that individuals are immunocompetent.

### Location of glycoprotein epitopes with high binding affinity to HLA Class II molecules

We found that more than one-half of high affinity binding epitopes were located within the first 500 AA of the glycoprotein (Figs. 6 and 7), with two hot spots in the interval S200-S400 AA. Therefore, vaccines containing epitopes from that part of the glycoprotein would have a high chance of good match with HLA Class II molecules, the first step in initiating antibody production. These findings are in contrast with a recent paper^3^ which described the most “immunodominant” epitopes in amino acid positions S510-S570 of the S protein, all of which have low affinities in our analysis (Figs. 6 and 7). Although the authors discuss the concept of immunodominance, it should be pointed out that effective immunogenicity which they assessed on T and B cells from mice is necessary but not sufficient. The reason lies in the fact that T cells are the first to interact with the antigen-HLA molecule complex, not the antigen alone: in the absence of such a match, even a highly antigenic epitope in vitro will not elicit antibody formation in vivo. Therefore, binding affinities of glycoprotein epitopes to HLA Class II molecules are crucial for effective vaccine development.

### Application to individuals and populations

An important aspect of this study focused on determining the ability of an individual for a good match between spike glycoprotein epitopes and their own HLA Class II molecules. Assuming that individuals are immunocompetent otherwise (i.e. they are not immunocompromised due to disease, age, or drug intake), the goodness of the match above is crucial in initiating antibody production because it the antigen epitope – HLA Class II molecule complex that activates CD4+ T lymphocytes. Now, every individual carries 2 from each of the 3 classical HLA Class II genes (DPB1, DQB1, DRB1), for a total of 6 such alleles. We combined that information with the binding affinity of each allele we determined in this study, to derive estimates of the “goodness of match” of an individual, and then extended this analysis to populations of individuals using a bootstrap procedure. There were three major findings from this analysis. First, the goodness of match of an individual can be estimated and ranked within the population, thus providing information concerning the rank of that individual among others with respect to that measure. This, in turn, will help identify individuals who may not be able to produce an adequate number of antibodies following SARS-CoV-2 infection or vaccination. Second, the goodness of match of a population overall can be estimated and used as a predictor of the expected antibody response in a natural setting (epidemic) or following vaccination. It should be noted that both cases above, regarding individuals and populations, rely on two factors, namely the HLA Class II genetic makeup and the binding affinity of an allele in that makeup. For an individual, both of these factors are known and, therefore, it is straightforward to compute the goodness of match. However, for a population, an additional critical element is the frequency of occurrence of an allele, information that has a major bearing on estimating the goodness of match in the population as a whole, since allele affinities will have to be weighted by their frequencies in the population (see equation 5 above). In the present study, we used 66 HLA Class II alleles with global frequencies ≥0.01. However, there are many more alleles with lower frequencies, and, even more important, the frequencies of various alleles differ across populations (countries, ethnicities, etc.). Unfortunately, complete lists of HLA Class II alleles are not available for any population because it has never been a useful goal to obtain such a list; instead, most information comes from studies related to organ transplantation. Be that as it may, here we outlined a rigorous procedure that will provide the population estimate of goodness of spike glycoprotein-HLA Class II needed: additional information about added alleles will naturally increase the accuracy of the estimate but will not affect the procedure.

### Association between allele frequency and binding affinity

We found a significant positive association between allele frequency and binding affinity, such that average higher allele frequencies (across 6 alleles of the HLA Class II genetic makeup) were associated with higher average binding affinities (Fig. 9). This suggests an evolutionary pressure such that alleles conferring higher protection against SARS-CoV-2 spike glycoprotein ultimately became more frequent. A further investigation of this relation in individual genes revealed a highly significant positive association between single allele frequency and binding affinity only for the DPB1 gene (Fig. 10). The source of this postulated evolutionary drive is unclear, since SARS-CoV-2 is a novel virus. A possible explanation would be that epitopes of the SARS-CoV-2 spike glycoprotein are shared with those of other proteins of virus that have been present for a long time. This could be a plausible explanation for the current observation that exposure to common cold (presumably caused by other corona viruses) may protect against COVID-19^19^. We are currently investigating in our lab this general idea by comparing the SARS-CoV-2 spike glycoprotein to proteins of other upper respiratory viruses, and evaluating their binding affinities to the same 66 alleles to find out a possible common association with alleles of DPB1. If this hypothesis is true, age-long upper respiratory epidemics may have provided the evolutionary pressure for selecting HLA Class II molecules with high binding affinity to such epitopes, thus conferring a survival advantage to their carriers.

### Relevance to the COVID-19 pandemic

A pivotal role of HLA Class II in the ability to mount antiSARS-CoV-2 antibodies was also indicated in a study of a cohort of convalescent patients who developed antibodies against different areas of the spike protein of the virus (S1, RBD, and S2 regions) with variable titers of antibodies. More specifically, ≈30% of recovered mild COVID-19 patients generated a deficient level of neutralizing antibody titers; in 10 of the 175 patients, the level was below the limit of detection while in contrast, two patients showed very high titers. This finding strongly suggests that the variable ability to mount antibodies to the virus should depend on HLA genotype, at least in part. The authors concluded that the interplay between virus and host immune response in coronavirus infections should be further explored for the development of effective vaccine against SARS-CoV-2^20^, a point addressed in detail herein.

As to the duration of anti-virus antibodies, these reached a maximum in the first 20 days of infection in a cohort of 70 patients who had variable antibody titers, being highest during day 31–40 since onset, and decreasing slightly afterwards^20^. Thus, changes of antibody levels and their duration were individual-specific. The authors concluded that the titers of antibodies and how long any immunity in patients will last is a key question to be addressed for safe and effective antiviral treatments and vaccines in the future^20^; this further indicates differences between individuals depending on their ability to mount and sustain an optimal antibody response pointing to the paramount significance of the HLA genotype.

Finally, the published information regarding different antibodies raised by COVID-19 patients strengthens the importance of our data which demonstrates specific S-domain virus peptides binding with high affinity to different HLA class II alleles. This information is critical for future vaccine development, which will serve for (1) strategic clinical management; (2) evaluation of vaccination efficiency in different individuals in the general population; (3) assignment of clinical professional and managerial teams interacting with COVID-19 patients^20^.

## Figures and Tables

**Table 1.** HLA Class II alleles used, ordered by their global frequencies in descending order. (See text for details.)

Allele	Frequency	Allele	Frequency	Allele	Frequency
DPB1*04:01	0.23022	DQB1*03:01	0.24528	DRB1*07:01	0.11305
DPB1*101:01	0.177	DQB1*02:01	0.13308	DRB1*15:01	0.0956
DPB1*05:01	0.16296	DQB1*03:02	0.10029	DRB1*03:01	0.0885
DPB1*04:02	0.16181	DQB1*05:01	0.0997	DRB1*11:01	0.07516
DPB1*02:01	0.15451	DQB1*06:02	0.07696	DRB1*01:01	0.06829
DPB1*03:01	0.0676	DQB1*02:02	0.07162	DRB1*13:01	0.05585
DPB1*01:01	0.05857	DQB1*03:03	0.05454	DRB1*11:04	0.05065
DPB1*13:01	0.04415	DQB1*04:02	0.05185	DRB1*04:01	0.0442
DPB1*14:01	0.04115	DQB1*06:01	0.05043	DRB1*13:02	0.03842
DPB1*02:02	0.02725	DQB1*05:02	0.04806	DRB1*16:01	0.03479
DPB1*09:01	0.02473	DQB1*05:03	0.0427	DRB1*14:01	0.03005
DPB1*17:01	0.02403	DQB1*06:03	0.04042	DRB1*14:54	0.02665
DPB1*28:01	0.01827	DQB1*06:04	0.02836	DRB1*15:02	0.02583
DPB1*77:01	0.01597	DQB1*04:01	0.02426	DRB1*12:01	0.02435
DPB1*11:01	0.0146	DQB1*06:09	0.01356	DRB1*04:04	0.02248
DPB1*18:01	0.01448			DRB1*09:01	0.02224
DPB1*107:01	0.014			DRB1*04:05	0.02144
DPB1*10:01	0.01295			DRB1*08:01	0.02097
DPB1*21:01	0.01172			DRB1*12:02	0.02014
DPB1*22:01	0.01149			DRB1*04:03	0.01807
DPB1*06:01	0.01001			DRB1*01:02	0.01745
				DRB1*13:03	0.0167
				DRB1*04:11	0.01642
				DRB1*08:03	0.01629
				DRB1*04:07	0.01524
				DRB1*16:02	0.01494
				DRB1*14:02	0.0148
				DRB1*10:01	0.01429
				DRB1*08:02	0.01421
				DRB1*04:02	0.01362

**Table 2.** LPR values for all alleles, ranked from smallest (highest binding affinity) to largest (lowest binding affinity). The associated AA sequence and its location along the spike glycoprotein are also given. See text for details.

Allele	LMR	Sequence for LMR	Start position	End position
DRB1*08:03	0.01	GTTLDSKTQSLLIVNNATNVVI	106	128
DRB1*14:02	0.01	GTTLDSKTQSLLIVNNATNVVI	106	128
DRB1*13:02	0.02	GTTLDSKTQSLLIVNNATNVVI	106	128
DPB1*04:02	0.04	NLCPFGEVFNATRFASVYAWNR	333	355
DPB1*77:01	0.04	NLCPFGEVFNATRFASVYAWNR	333	355
DRB1*04:11	0.05	LPIGINITRFQTLLALHRSYLT	228	250
DRB1*01:02	0.07	PIGINITRFQTLLALHRSYLTP	229	251
DRB1*04:03	0.07	TQQLIRAAEIRASANLAATKMS	1008	1030
DPB1*02:01	0.08	LCPFGEVFNATRFASVYAWNRK	334	356
DPB1*01:01	0.09	CPFGEVFNATRFASVYAWNRKR	335	357
DPB1*04:01	0.09	TNLCPFGEVFNATRFASVYAWN	332	354
DRB1*04:04	0.1	LPIGINITRFQTLLALHRSYLT	228	250
DPB1*02:02	0.11	NLCPFGEVFNATRFASVYAWNR	333	355
DPB1*10:01	0.11	YVTQQLIRAAEIRASANLAATK	1006	1028
DPB1*101:01	0.11	NLCPFGEVFNATRFASVYAWNR	333	355
DPB1*14:01	0.11	YVTQQLIRAAEIRASANLAATK	1006	1028
DPB1*17:01	0.11	YVTQQLIRAAEIRASANLAATK	1006	1028
DRB1*15:01	0.11	PIGINITRFQTLLALHRSYLTP	229	251
DPB1*06:01	0.13	CPFGEVFNATRFASVYAWNRKR	335	357
DPB1*18:01	0.14	PFGEVFNATRFASVYAWNRKRI	336	358
DQB1*03:01	0.14	LAGTITSGWTFGAGAALQIPFA	877	899
DRB1*12:02	0.14	LPIGINITRFQTLLALHRSYLT	228	250
DPB1*28:01	0.16	LPIGINITRFQTLLALHRSYLT	228	250
DRB1*04:02	0.16	PIGINITRFQTLLALHRSYLTP	229	251
DPB1*09:01	0.17	PFGEVFNATRFASVYAWNRKRI	336	358
DPB1*107:01	0.17	PFGEVFNATRFASVYAWNRKRI	336	358
DPB1*13:01	0.17	PFGEVFNATRFASVYAWNRKRI	336	358
DQB1*06:01	0.17	LTPGDSSSGWTAGAAAYYVGYL	248	270
DPB1*03:01	0.18	VTQQLIRAAEIRASANLAATKM	1007	1029
DPB1*05:01	0.18	LPIGINITRFQTLLALHRSYLT	228	250
DPB1*11:01	0.18	LCPFGEVFNATRFASVYAWNRK	334	356
DRB1*16:01	0.18	PIGINITRFQTLLALHRSYLTP	229	251
DRB1*10:01	0.19	VASQSIIAYTMSLGAENSVAYS	686	708
DRB1*08:02	0.2	VTQQLIRAAEIRASANLAATKM	1007	1029
DRB1*15:02	0.21	LDSKTQSLLIVNNATNVVIKVC	109	131
DPB1*22:01	0.22	PFGEVFNATRFASVYAWNRKRI	336	358
DRB1*12:01	0.22	LPIGINITRFQTLLALHRSYLT	228	250
DRB1*13:03	0.22	LDSKTQSLLIVNNATNVVIKVC	109	131
DRB1*04:07	0.25	DSKTQSLLIVNNATNVVIKVCE	110	132
DRB1*14:01	0.27	LPIGINITRFQTLLALHRSYLT	228	250
DRB1*14:54	0.27	LPIGINITRFQTLLALHRSYLT	228	250
DQB1*06:02	0.29	ITSGWTFGAGAALQIPFAMQMA	881	903
DQB1*06:03	0.29	ITSGWTFGAGAALQIPFAMQMA	881	903
DPB1*21:01	0.3	VTQQLIRAAEIRASANLAATKM	1007	1029
DQB1*03:03	0.3	TSGWTFGAGAALQIPFAMQMAY	882	904
DRB1*09:01	0.31	GWTFGAGAALQIPFAMQMAYRF	884	906
DQB1*04:01	0.34	GWTFGAGAALQIPFAMQMAYRF	884	906
DQB1*04:02	0.34	GWTFGAGAALQIPFAMQMAYRF	884	906
DRB1*08:01	0.44	VGGNYNYLYRLFRKSNLKPFER	444	466
DRB1*11:01	0.44	VGGNYNYLYRLFRKSNLKPFER	444	466
DRB1*04:05	0.47	LPIGINITRFQTLLALHRSYLT	228	250
DRB1*16:02	0.52	QDLFLPFFSNVTWFHAIHVSGT	51	73
DQB1*03:02	0.54	TSGWTFGAGAALQIPFAMQMAY	882	904
DQB1*06:09	0.64	GWTFGAGAALQIPFAMQ-MAYRF	884	906
DRB1*11:04	0.66	VGGNYNYLYRLFRKSNLKPFER	444	466
DQB1*06:04	0.8	GWTFGAGAALQIPFAMQ-MAYRF	884	906
DRB1*04:01	0.92	QQLIRAAEIRASANLAATKMSE	1009	1031
DRB1*03:01	1.1	KHTPINLVRDLPQGFSALEPLV	205	227
DRB1*07:01	1.1	LPIGINITRFQTLLALHRSYLT	228	250
DRB1*01:01	1.2	PIGINITRFQTLLALHRSYLTP	229	251
DRB1*13:01	1.7	LPIGINITRFQTLLALHRSYLT	228	250
DQB1*05:02	2.5	CNGVEGFNCYFPLQSYGFQPTN	479	501
DQB1*05:03	2.6	GVEGFNCYFPLQSYGFQPTNGV	481	503
DQB1*05:01	2.8	VSPTKLNDLCFTNVYADSFVIR	381	403
DQB1*02:01	3.9	INLVRDLPQGFSALEPLVDLPI	209	231
DQB1*02:02	3.9	INLVRDLPQGFSALEPLVDLPI	209	231

**Table 3.** PR5 values for all alleles, ranked from highest (best affinity) to lowest. See text for details.

Allele	LE5MR
DRB1*04:05	9.83213
DRB1*04:01	9.27258
DRB1*04:07	8.39329
DRB1*13:02	8.39329
DRB1*10:01	8.31335
DRB1*15:02	8.07354
DRB1*16:02	8.07354
DPB1*04:01	7.83373
DPB1*02:02	7.67386
DPB1*101:01	7.67386
DPB1*02:01	7.35412
DPB1*04:02	7.19424
DPB1*77:01	7.19424
DRB1*13:03	7.11431
DQB1*06:01	7.03437
DRB1*16:01	6.79456
DPB1*01:01	6.63469
DRB1*04:04	6.63469
DQB1*06:02	6.31495
DRB1*07:01	6.31495
DPB1*28:01	6.15508
DPB1*22:01	5.9952
DRB1*14:02	5.9952
DQB1*03:01	5.83533
DQB1*04:01	5.7554
DQB1*04:02	5.7554
DRB1*01:01	5.7554
DRB1*15:01	5.7554
DRB1*04:11	5.59552
DQB1*06:03	5.51559
DRB1*04:03	5.51559
DPB1*09:01	5.35572
DPB1*107:01	5.35572
DPB1*13:01	5.35572
DPB1*06:01	5.27578
DPB1*18:01	5.19584
DRB1*09:01	5.19584
DPB1*03:01	5.11591
DPB1*05:01	5.11591
DPB1*11:01	4.71623
DPB1*21:01	4.71623
DPB1*10:01	4.63629
DPB1*14:01	4.63629
DPB1*17:01	4.63629
DQB1*03:03	4.47642
DRB1*08:03	3.67706
DRB1*14:01	3.67706
DRB1*14:54	3.67706
DRB1*04:02	3.51719
DRB1*08:01	3.51719
DRB1*12:01	3.43725
DQB1*06:04	3.35731
DRB1*11:01	3.27738
DRB1*01:02	3.11751
DRB1*12:02	3.11751
DQB1*06:09	2.95763
DQB1*03:02	2.79776
DRB1*11:04	2.71783
DRB1*08:02	2.55795
DQB1*05:03	1.91847
DRB1*03:01	1.91847
DRB1*13:01	1.83853
DQB1*05:02	1.43885
DQB1*05:01	0.79936
DQB1*02:01	0.39968
DQB1*02:02	0.39968

## References

[1] Padron-Regalado E. Vaccines for SARS-CoV-2: Lessons from other coronavirus strains. Infect Dis Ther. 2020; 9(2): 1–20. 32328406 10.1007/s40121-020-00300-x PMC7177048

[2] Jaimes JA, André NM, Chappie JS, Phylogenetic analysis and structural modeling of SARS-CoV-2 spike protein reveals an evolutionay distinct and proteolytically sensitive activation loop. J Mol Biol. 2020; 432 (10): 3309–3325. 32320687 10.1016/j.jmb.2020.04.009 PMC7166309

[3] Zhang BZ, Hu YF, Chen LL, Mining of epitopes on spike protein of SARS-CoV-2 from COVID-19 patients. Cell Res. 2020; 30(8): 702–704. doi: 10.1038/s41422-020-0366-x. 32612199 PMC7327194

[4] Blum JS, Wearsch PA, Cresswell P. Pathways of antigen processing. Annu. Rev. Immunol 2013; 31: 443–473. 23298205 10.1146/annurev-immunol-032712-095910 PMC4026165

[5] Straube F. Antigen presentation via MHC Class II molecules. In: Vohr HW. (eds) Encyclopedic Reference of Immunotoxicology. Springer Berlin Heidelberg. 2005. 10.1007/3-540-27806-0_99

[6] Hanna S, Etzioni A. MHC class I and II deficiencies. J Allergy Clin Immunol. 2014; 134: 269–275. 25001848 10.1016/j.jaci.2014.06.001

[7] Wrapp D, Nianshuang Wang N, Corbett KS, Cryo-EM Structure of the 2019-nCoV spike in the prefusion conformation. Science. 2020; 367(6483): 1260–1263. 32075877 10.1126/science.abb2507 PMC7164637

[8] Walls AC, Park YJ, Tortorici MA, structure, function, and antigenicity of the SARS-CoV-2 spike glycoprotein. Cell. 2020; 180: 281–292. 10.1016/j.cell.2020.02.058 PMC7102599 32155444

[9] Wang Q, Zhang Y, Wu L, structural and functional basis of SARS-CoV-2 entry by using human ACE2. Cell. 2020; 181: 1–11. 32275855 10.1016/j.cell.2020.03.045 PMC7144619

[10] Ou X, Liu Y, Lei X, Characterization of spike glycoprotein of SARS-CoV-2 on virus entry and its immune cross-reactivity with SARS-CoV. Nature Communications. 2020; 11: 1620. | 10.1038/s41467-020-15562-9 | www.nature.com/naturecommunications PMC7100515 32221306

[11] The Allele Frequence Database. http://www.allelefrequencies.net/ Accessed on 6.16.2020.

[12] UniProt Consortium. UniProt: A Worldwide Hub of Protein Knowledge Nucleic Acids Res. 2019; 47(D1):D506–D515. 30395287 10.1093/nar/gky1049 PMC6323992

[13] Charonis S, James LM, Georgopoulos AP. In silico assessment of binding affinities of three dementia-protective Human Leukocyte Antigen (HLA) alleles to nine human herpes virus antigens. Curr Res Transl Med. 2020; 68(4): 211–216. doi: 10.1016/j.retram.2020.06.002. 32624427

[14] Immune Epitope Database and Analysis Resource: www.iedb.org. Accessed on 6.16.2020.

[15] Jensen KK, Andreatta M, Marcatili P, Improved methods for predicting peptide binding affinity to MHC class II molecules. Immunology. 2018; 154(3): 394–406. doi:10.1111/imm.12889. 29315598 PMC6002223

[16] Groß A, Hashimoto CH, Sticht H, Eichler J. Synthetic peptides as protein mimics. Front Bioeng Biotechnol. 2015; 3: article 211. doi: 10.3389/fbioe.2015.00211 PMC4717299 26835447

[17] Sachdeva S, Joo H, Tsai J, A rational approach for creating peptides mimicking antibody binding. Scientific Reports volume 9, Article number: 997. 2019 10.1038/s41598-018-37201-6 30700733 PMC6353898

[18] Mateus J, Grifoni A, Tarke A, Selective and cross-reactive SARS-CoV-2 T cell epitopes in unexposed humans. Science. 2020. 10.1126/science.abd3871 PMC7574914 32753554

[19] Shi Y, Wang Y, Shao C, COVID-19 infection: the perspectives on immune responses. Cell Death Differ. 2020; 27(5): 1451–1454. doi: 10.1038/s41418-020-0530-3. 32205856 PMC7091918

[20] Wang X, Guo X, Qianqian Xin O, Neutralizing Antibodies Responses to SARS-CoV-2 in COVID-19 Inpatients and Convalescent Patients Clin Infect Dis. 2020 Jun 4; ciaa721. doi: 10.1093/cid/ciaa721. Online ahead of print. PMC7314147 32497196

